# Diagnostic accuracy of circulating free DNA testing for the detection of KRAS mutations in non-small cell lung cancer: A systematic review and meta-analysis

**DOI:** 10.3389/fgene.2022.1015161

**Published:** 2022-10-25

**Authors:** Maria Palmieri, Elisabetta Zulato, Sissel Gyrid Freim Wahl, Nicolas Guibert, Elisa Frullanti

**Affiliations:** ^1^ Med Biotech Hub and Competence Center, Department of Medical Biotechnologies, University of Siena, Siena, Italy; ^2^ Basic and Translational Oncology Unit, Istituto Oncologico Veneto IOV—IRCCS, Padova, Italy; ^3^ Department of Pathology, St. Olavs Hospital, Trondheim University Hospital, Trondheim, Norway; ^4^ Department of Clinical and Molecular Medicine, NTNU, Norwegian University of Technology and Science, Trondheim, Norway; ^5^ Thoracic Oncology Department, Larrey Hospital, University Hospital of Toulouse, Toulouse, France; ^6^ Inserm, Centre de Recherche en Cancérologie de Toulouse, CRCT UMR-1037, Toulouse, France; ^7^ University of Toulouse III (Paul Sabatier), Toulouse, France

**Keywords:** cancer risk, diagnostic value, genetic biomarkers, single-nucleotide polymorphisms, liquid biopsy

## Abstract

Kirsten rat sarcoma viral oncogene homolog (KRAS) gene encodes a GTPase that acts as a molecular switch for intracellular signal transduction, promoting cell growth and proliferation. Mutations in the KRAS gene represent important biomarkers for NSCLC targeted therapy. However, detection of KRAS mutations in tissues has shown some limitations. During the last years, analyses of circulating free DNA (cfDNA) has emerged as an alternative and minimally invasive, approach to investigate tumor molecular changes. Here, we assessed the diagnostic performance of cfDNA analysis, compared to tissues through a meta-analysis and systematic review of existing literature. From 561 candidate papers, we finally identified 40 studies, including 2,805 NSCLC patients. We extracted values relating to the number of true-positive, false-positive, false-negative, and true-negative. Pooled sensitivity, specificity, positive likelihood ratio, negative likelihood ratio, and diagnostic odds ratio, each with 95% CI, were calculated. A summary receiver operating characteristic curve and the area under curve (AUC) were used to evaluate the overall diagnostic performance. The pooled sensitivity was 0.71 (95% CI 0.68–0.74) and the specificity was 0.93 (95% CI 0.92–0.94). The diagnostic odds ratio was 35.24 (95% CI 24.88–49.91) and the area under the curve was 0.92 (SE = 0.094). These results provide evidence that detection of KRAS mutation using cfDNA testing is of adequate diagnostic accuracy thus offering to the clinicians a new promising screening test for NSCLC patients.

## Introduction

Lung cancer (LC) is the leading cause of cancer-related mortality worldwide representing 18% of the total cancer deaths in 2020 (Sun et al., 2021). Non-small cell lung cancer (NSCLC), the largest group of LC, accounts for approximately 80% of new cases. These patients are often unsuitable for curative surgery duo to the advanced stage at the time of diagnosis (Ganti et al., 2021).

In the last decade, one of the most exciting advances in medical oncology is the application of personalized treatment tailored to the patient’s genetic background, primarily based on mutations in the epidermal growth factor receptor (EGFR) and in the Kirsten rat sarcoma viral oncogene homolog (KRAS) genes (Qiu et al., 2015; Hames et al., 2016). KRAS gene encodes a small GTPase that functions as an intracellular signaling protein promoting cell growth and proliferation (King et al., 2013). Approximately 30% of adenocarcinomas, the most common histological subtype of NSCLC, harbor mutations of the KRAS gene. Mutations in KRAS gene lead to oncogenic conversion ensuing in constitutive activation of downstream signal transduction cascades and thus cancer development and progression as well as specific drug sensitivity. KRAS mutations in NSCLC predominantly occur at codon 12 or codon 13 (Ghimessy et al., 2029) and represent an important biomarker for NSCLC therapy. Moreover, recently, new therapeutic agents (i.e., adagrasib and sotorasib) that target specifically the KRAS G12C variant have been developed and shown promising results in both preclinical and clinical trials (Skoulidis et al., 2021).

The gold standard for detecting cancer mutations has been based on molecular testing of tumor biopsies. However, obtaining a tumor biopsy requires invasive techniques, and is not suitable for real-time monitoring of cancer mutations. Furthermore, it is often difficult to obtain sufficient tissues for molecular testing through biopsy (Overman et al., 2013). During the last years, in order to answer the need of a more accessible and sequentially repeatable approach for tumor genetic analysis, “liquid biopsy” has emerged. The most successful use of liquid biopsy is the analysis of tumor DNA fragments that are released into the bloodstream through apoptosis, necrosis, and/or active secretion processes of cancer cells in the form of circulating free DNA (cfDNA) (Diehl et al., 2008). This approach turned out to be a minimally invasive and efficient method to investigate cancer cells which enables taking multiple blood samples over time (serial sampling) and thus informing about what kind of molecular changes are taking place in the tumor (Castro-Giner et al., 2018).

To date, many meta-analyses have investigated the performance of cfDNA in detecting EGFR mutation status in patients with NSCLC and only two meta-analyses evaluated also KRAS (Fan et al., 2017; Chen et al., 2020). However, the diagnostic accuracy of cfDNA testing for the detection of KRAS mutations remains controversial and not conclusive since the results vary among these two meta-analyses justifying further investigation.

Herein, we conducted a comprehensive systematic review and meta-analysis of available studies that compare the concordance between results on cfDNA within liquid biopsy and genomic DNA within tumor tissue to obtain an all-inclusive evaluation of the diagnostic accuracy of cfDNA testing for detection of KRAS mutations in NSCLC patients.

## Materials and methods

### Search strategy, inclusion and exclusion criteria and data extraction

We systematically searched the PubMed, Medline, Embase and Web of Science databases up to 7 July 2022 for studies reporting the diagnostic performance of cfDNA comparing sensitivity and specificity between tissue and blood in detecting *KRAS* mutations in NSCLC patients, using different combinations of the keywords: ‘‘lung neoplasms’’ or ‘‘lung cancer’’ or “NSCLC”, “*KRAS*,” “re-biopsy” or “repeat biopsy” or “liquid biopsy,” ‘‘serum’’ or ‘‘plasma’’ or ‘‘circulating,’’ and ‘‘mutations,’’ without any restriction. Abbreviations and alternative spellings and were also considered. Eligible publications were evaluated by checking titles and abstracts.

The references of all computer-identified publications were also checked for identifying additional studies that might have been missed in the initial search. Relevant reviews were also manually searched. Publications were checked for overlapping patient populations and, in the case of multiple publications from the same research group on overlapping cohorts, only the largest or most recent study was selected.

All studies evaluating sensitivity and specificity between tissue biopsy and liquid biopsy in *KRAS*-mutated NSCLC were considered eligible for the meta-analysis. The inclusion criteria were: 1) all NSCLC patients involved should be diagnosed cytologically or histopathologically; 2) tissue and blood biopsies should be paired in the same patient; 3) KRAS mutation status should be detected by circulating cell free DNA and verified in tumor tissues; 4) enough reported data to construct the diagnostic 2 × 2 table. In the process of assessing the eligibility of the studies, only articles written in English were included. We subsequently excluded studies that involved cell lines or artificial samples or where cfDNA was not detected. Finally, articles were excluded if they presented data in a way that did not allow it to be extracted properly (e.g., studies with mixed data from different types of cancers other than NSCLC).

Information collected from all eligible articles included the study characteristics (authors’ names, publication date and journal, country of study, number of patients); the clinical data (histological analysis, clinical stage); the results (method for tissue and blood biopsy; number of true positives (TP), true negatives (TN), false positives (FP), and false negatives (FN) between tissue and blood, and concordance between the two methods).

The original data from the eligible studies reporting partial information on TP, TN, FP, and FN, in comparison to sensitivity and specificity between tissue and cfDNA analysis were obtained by contacting the corresponding authors. Data on individual patients were collected by requesting the completion of a standardized form. The sensitivity equals [TP / (TP + FN)], while specificity equals [TN / (TN + FP)]. If not present, we computed the concordance rate as [(TP + TN)/n]. All records were reviewed and checked by two authors independently (MP and EF) and reached consensus at each eligible study.

### Statistical analyses

Meta-analysis was carried out using the Rev-Man v.5.4 (provided by The Cochrane Collaboration, Oxford, England), Meta-DiSc and R packages (version 4.2.1). The combined sensitivity, specificity, positive likelihood ratio (PLR = sensitivity/(1-specificity)), negative likelihood ratio (NLR = (1-sensitivity)/specificity), positive predicted value, negative predicted value, diagnostic odds ratio (DOR = PLR/NLR) and corresponding 95% confidence intervals (95% CI) were calculated by the accuracy data (TP, TN, FP, and FN) collected from each studies. Based on these data, the summary receiver operating characteristic (SROC) was created and the area under the curve (AUC) was calculated.

A random-effects model, fitted *via* the general linear (mixed-effects) model, was used for all analyses, recognizing that its use can reduce the effect of larger studies and minimize the possible presence of heterogeneity among the studies accounting for the variation both within a study or between the many different studies included in the meta-analysis. Homogeneity of study results in different groupings was assessed using the *Q* and *I* ^2^ statistic. Publication bias was estimated by visual inspection of funnel plots, and a *p* value <0.05 indicated the occurrence of publication bias. Spearman correlation coefficient and *p* value were calculated to assess the threshold effect.

## Results

### Study selection

**FIGURE 1:**
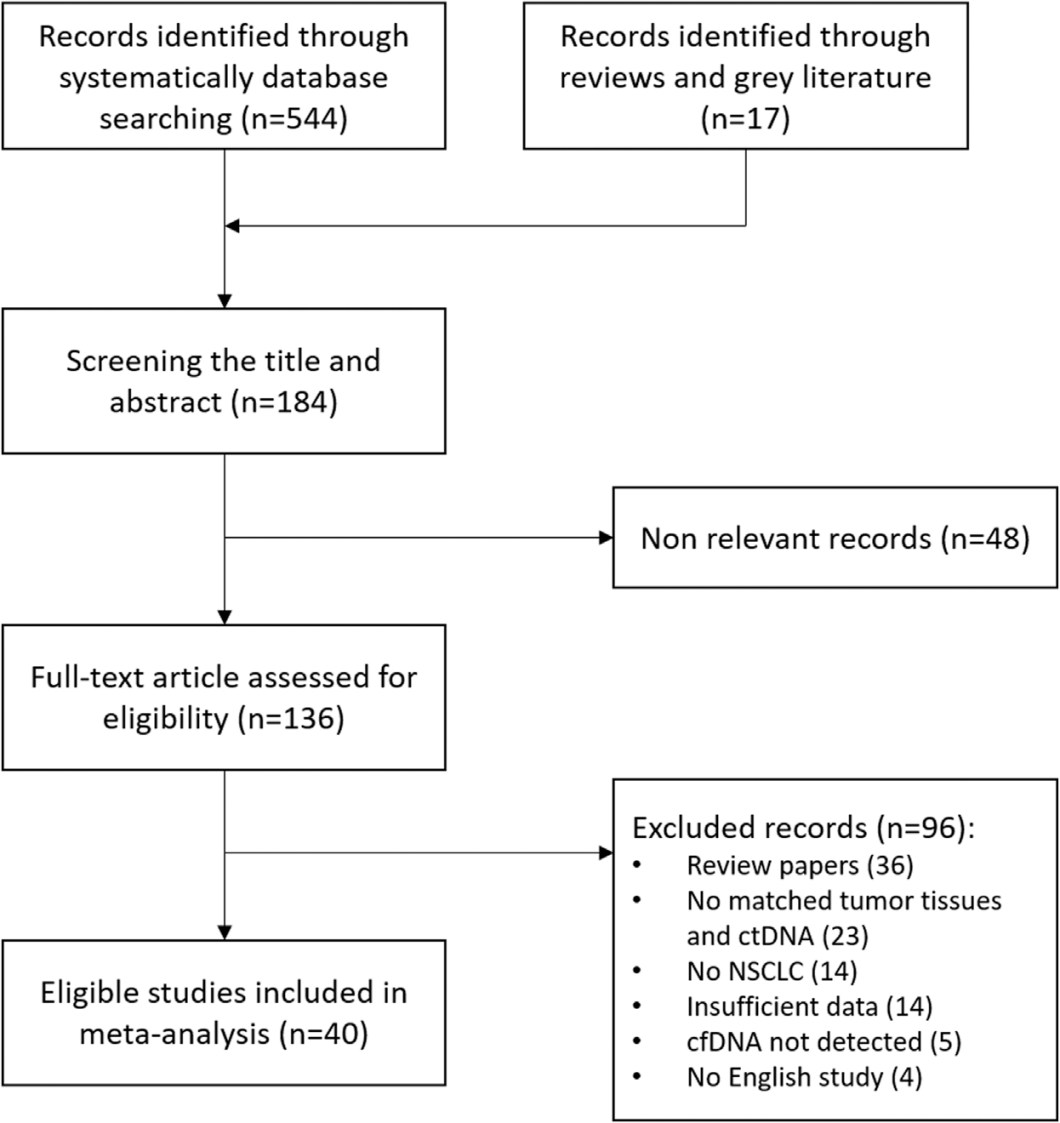
Flow diagram of literature screening and study selection.

A total of 561 potential studies were initially evaluated for the meta-analysis on the results of the bibliographic search. After primary screening checking titles and abstracts, 136 full-text articles were selected for further evaluation of eligibility and scanned rigorously in full text. The main reasons for exclusion were reviews, not human studies and incorrect or mixed tumor type. After exclusion of studies, a total of 40 eligible studies were identified and finally included in our meta-analysis, comprising 2,805 NSCLC cases. A flowchart of the literature selection is shown in Figure 1.

### Characteristics of eligible studies

The forty-one eligible studies for meta-analysis were published between 2007 and 2022 and included 2,805 NSCLC patients from 13 countries. The mean number of patients for each study was 70 (range 8–200). Notably, the majority of studies were prospective (28/40) and only 12 out of 40 were retrospective. Various types of methods have been applied for the detection of *KRAS* mutation in cfDNA, and the targeted NGS sequencing was the most common method (21/40). The median age was 62.8 years (range 20–100), 54.4% of patients were male and 69.7% had a history of smoking (former or current). Most of the patients were at advanced stage (TNM III–IV). The main characteristics of the 40 included studies are shown in Table 1.

**TABLE 1:** Characteristics of eligible studies included in the meta- and pooled-analyses.

References	No. of cases	Country	Study design	Males/Females	Median age (range)	% Smoker (former or current)	NSCLC stage (I-IV)	Specimen type for ctDNA	Detection assay in ctDNA
Gautschi et al. (2007)	15	Switzerland	Pro	125/55	61 (36–81)	69	I-IV	Plasma	RFLP-PCR and Sequencing
Wang et al. (2010)	120	China	Pro	158/115	NA	41.4	IIIB, IV	Plasma	RFLP-PCR
Narayan A et al. (2012)	21	United States	Retro	NA	NA	NA	I-IV	Plasma	Targeted NGS
Punnoose EA et al. (2012)	25	United States, Australia	Retro	21/16	NA	83.8	IV	Plasma	In house TaqMan Assay
Nygaard et al. (2013)	10	Denmark	Pro	151/95	66 (40–80)	NA	II-IV	Plasma	ARMS-qPCR
Oxnard GR et al. (2014)	31	United States	Retro	NA	NA	NA	NA	Plasma	ddPCR
Couraud S et al. (2014)	68	France	Pro	13/93	NA	0	I-IV	Plasma	Targeted NGS
Freidin MB et al. (2015)	82	United Kingdom	Pro	52/41	NA	NA	I-IV	Plasma	Cold PCR-HMR
Guibert N et al. (2016)	32	France	Pro	21/11	NA	NA	IV	Plasma	ddPCR
Guo N et al. (2016)	41	China	Pro	22/19	52 (38–73)	NA	I-IV	Plasma	Targeted NGS
Paweletz C et al. (2016)	48	United States	Pro	19/29	57	NA	IV	Plasma	Targeted NGS
Thompson JC et al. (2016)	50	United States	Pro	33/69	64 (34–85)	50	II-IV	Plasma	Paired-end sequencing
Chen KZ et al. (2016)	58	China	Retro	33/25	64 (40–84)	37.9	I-II	Plasma	Targeted NGS
Sacher AG et al. (2016)	87	United States	Pro	68/112	62	NA	IIIB, IV	Plasma	ddPCR
Pécuchet N et al. (2016)	109	France	Pro	49/60	NA	67	III-IV	Plasma	Ultradeep-targeted NGS
Han JY et al. (2016)	135	South Korea	Pro	136/72	58 (29–82)	63	III-IV	Plasma	PNA champ-assisted melting curve
Del Re M et al. (2017)	8	Italy	Retro	13/20	62 (41–75)	33.3	III-IV	Plasma	ddPCR
Yao T et al. (2017)	39	China	Retro	19/20	62 (28–78)	25.6	IIIa-IV	Plasma	Targeted NGS
Mellert et a. (2017)	42	United States	Pro/Retro	NA	NA	NA	NA	Plasma	ddPCR
Wang Z et al. (2017)	103	China	Pro	48/55	64 (21–87)	32	III-IV	Plasma	cSMART
Wang X et al. (2017)	200	China	Pro	138/62	57 (NA)	NA	NA	Urine	ddPCR
Garcia J et al. (2018)	20	France	Retro	NA	NA	NA	IV	Plasma	Targeted NGS
Liu L et al. (2018)	72	China	Pro	44/28	59 (40–83)	40.3	IIIa-IV	Plasma	Targeted NGS
Papadopoulou E et al. (2019)	36	Greece	Pro	82/39	NA	NA	NA	Plasma	Targeted NGS
Tran LS et al. (2019)	40	Vietnam	Pro	33/25	62 (37–90)	43	IIIB, IV	Plasma	ddPCR and Ultradeep-targeted NGS
Leighl NB et al. (2019)	89	United States	Pro	129/153	69 (26–100)	76.2	IIIB, IV	Plasma	Targeted NGS
Remon J et al. (2019)	104	France	Pro	126/88	NA	83.3	IIIB, IV	Plasma	Targeted NGS
Li BT et al. (2019)	110	United States	Pro	47/80	66 (23–85)	NA	IIIB, IV	Plasma	Targeted NGS
Pritchett MA et al. (2019)	147	United States	Pro	84/94	NA	86.6	IIIB, IV	Plasma	Targeted NGS
Cho MS et al. (2020)	36	South Korea	Pro	25/11	66 (33–81)	NA	I-IV	Plasma	PANAmutyper™
Jiang J et al. (2020)	47	Germany	Retro	45/26	62 (NA)	85	IIIa-IV	Plasma	Targeted NGS
Zulato E et al. (2020)	58	Italy	Pro	31/27	68 (61–73)	86.2	NA	Plasma	ddPCR
Michaelidou K et al. (2020)	96	Greece	Pro	99/22	NA	88.6	III-IV	Plasma	ddPCR
Mehta A et al. (2021)	21	India	Retro	14/7	54 (28–79)	47.62	IIIB, IV	Plasma	Targeted NGS
Qvick A et al. (2021)	24	Sweden	Pro	32/28	72 (39–85)	80	I-IV	Plasma	Targeted NGS
Wahl SGF et al. (2021)	60	Norway	Retro	23/37	69 (47–83)	100	I-IV	Plasma	ddPCR
Lam VK et al. (2021)	76	United States	Retro	66/78	64 (28–96)	NA	I-IV	Plasma	Targeted NGS
Jiao XD et al. (2021)	185	China	Pro	110/75	64 (24–84)	NA	IIIB, IV	Plasma	Targeted NGS
Xie X et al. (2022)	71	China	Pro	54/16	60 (20–79)	46.5	I-IV	Sputum	Targeted NGS
Bauml JM et al. (2022)	189	United States	Pro	63/63	63.5 (56–70)	92.9	I-IV	Plasma	Targeted NGS
Total of cases	2,805								

NA, not available; Pro, prospective study; Retro, retrospective study; RFLP, Restriction Fragment Length Polymorphism; PCR, Polymerase Chain Reaction; NGS, Next Generation Sequencing; ddPCR, Droplet Digital PCR; ARMS-PCR, Amplification Refractory Mutation System PCR; PCR-HMR, High-resolution Melting PCR; PNA, Peptide Nucleic Acid; cSMART, circulating single-molecule amplification and re-sequencing technology; PANAmutyperTM, PNA Clamping-assisted Fluorescence Melting Curve Analysis.

### Diagnostic accuracy

**FIGURE 2:**
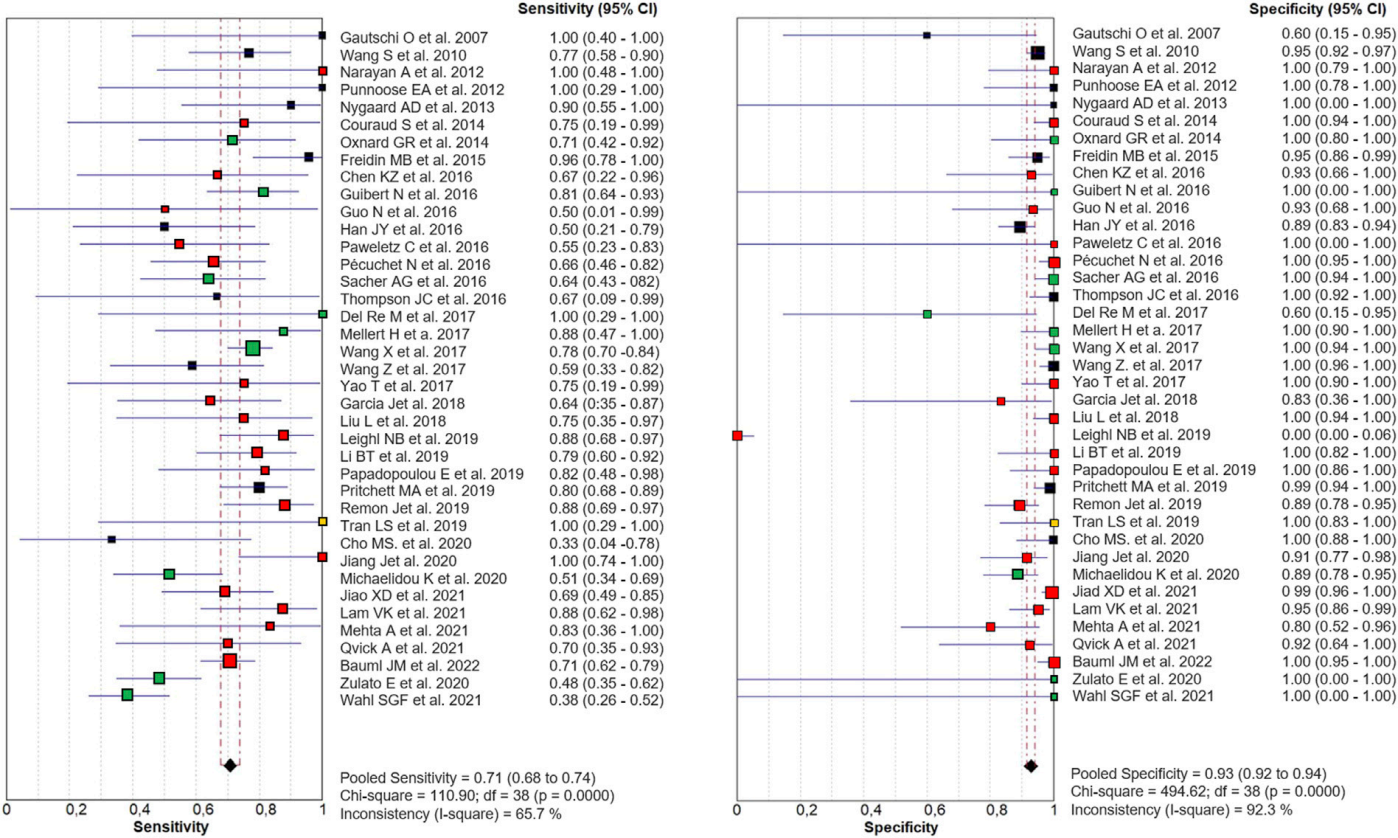
Paired Forest Plot of sensitivity and specificity of cfDNA testing in detecting KRAS mutation in NSCLC for 39 studies in the meta-analysis. Random-effects (RE) model used. The square and horizontal bars represented study-specific sensitivity and specificity and 95% confidence interval (CI). Squares in different colors represent a different diagnostic method (red = Targeted NSG; blue = ddPCR; yellow = both; black = other method). Diamonds represented the overall results. The pooled sensitivity was 0.71 (95% CI 0.68–0.74) and the specificity was 0.93 (95% CI 0.92–0.94).

Out of 40 eligible papers, 1 was excluded from our meta-analysis (Xie et al., 2022) since only TP and FN were reported in published data. The diagnostic accuracy of cfDNA analysis for the detection of *KRAS* mutations in NSCLC, compared with tumor tissue was shown in the Forest Plot for 39 studies, including 2,666 NSCLC patients. The combined sensitivity and specificity in the meta-analysis were 0.71 (95% CI 0.68–0.74) and 0.93 (95% CI 0.92–0.94), respectively (Figure 2).

**FIGURE 3:**
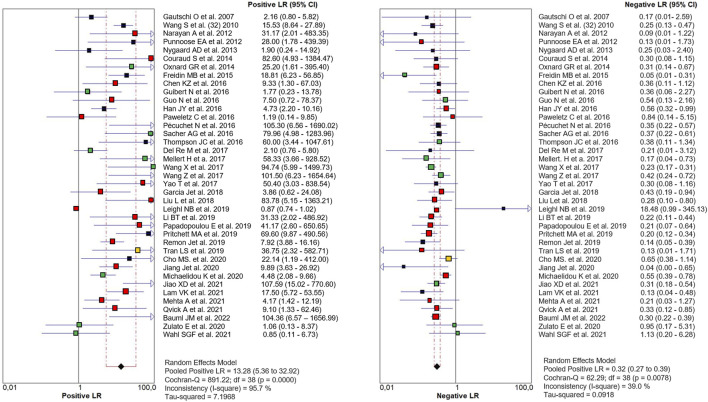
Paired Forest plot of PLR and NLR of cfDNA testing in detecting KRAS mutation in NSCLC for 39 studies in the meta-analysis. Random-effects (RE) model used. The square and horizontal bars represented study-specific PLR and NLR and 95% confidence interval (CI). Squares in different colors represent a different diagnostic method (red = Targeted NSG; blue = ddPCR; yellow = both; black = other method). Diamonds represented the overall results. The pooled PLR and NLR were 8.32 (95% CI 6.93–9.99) and 0.29 (95% CI 0.26–0.33), respectively.

**FIGURE 4:**
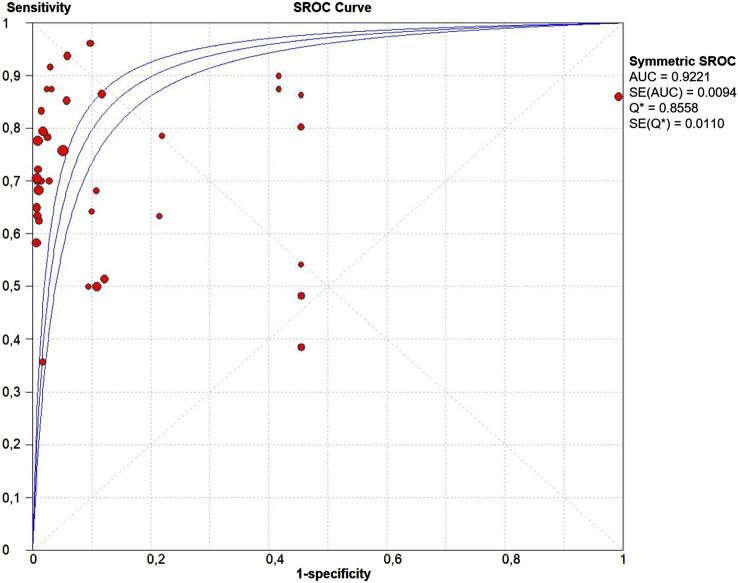
The summary receiver operating characteristic (SROC) curve. Points represent the pair of sensitivity and specificity at a given threshold for each study. The area under the curve was 0.92 (SE = 0.094).

The PLR and NLR of cfDNA were 8.32 (95% CI 6.93–9.99) and 0.29 (95% CI 0.26–0.33), respectively in the meta-analysis (Figure 3). The DOR was 35.24 (95% CI 24.88–49.91). Figure 4 shows the SROC plot with AUC of 0.92 (SE = 0.0094), indicating a high diagnostic accuracy of cfDNA test.

### Heterogeneity and publication bias

**FIGURE 5:**
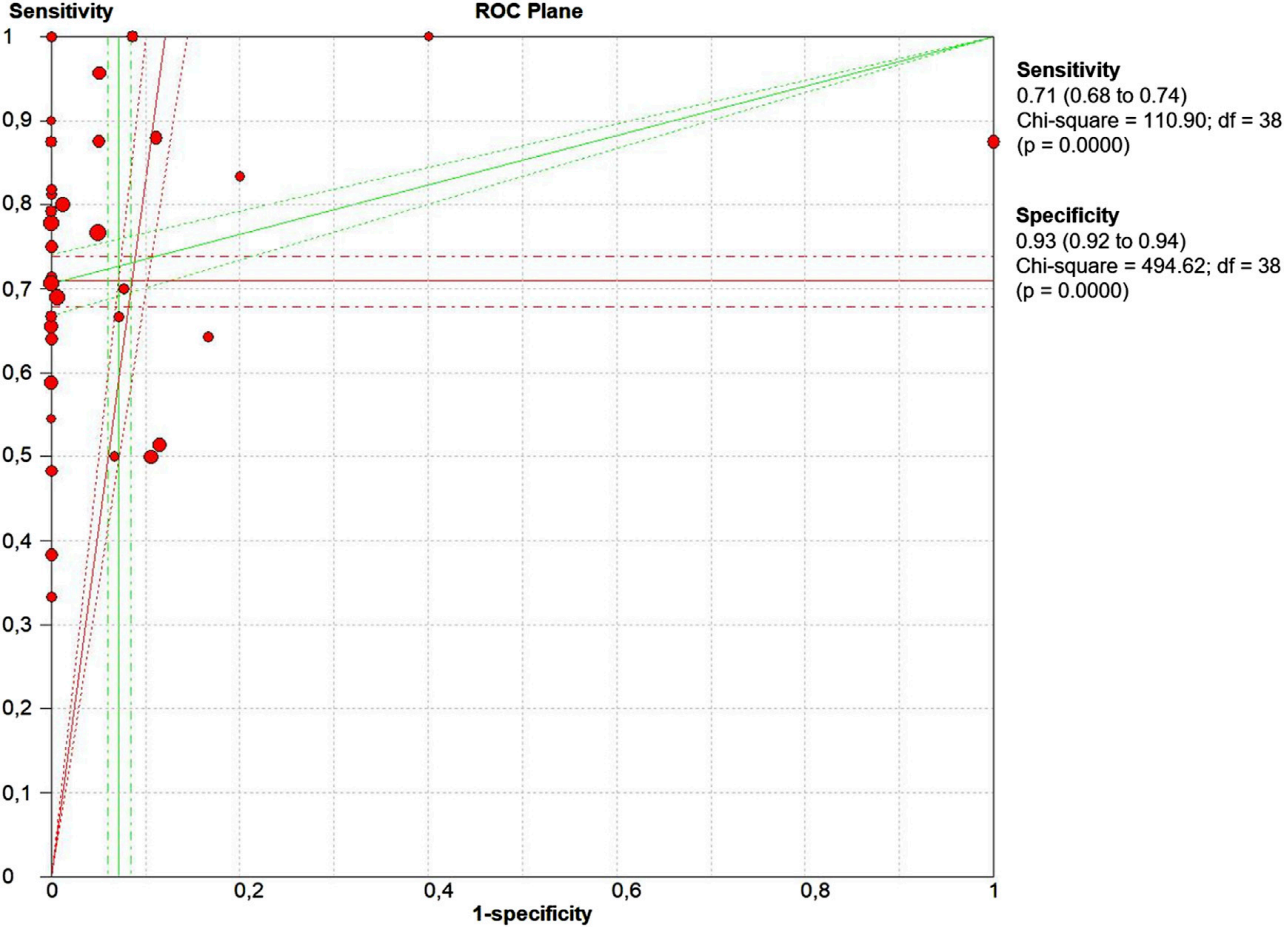
The receiver operative curve (ROC) plane plot. Points represent the pair of sensitivity and specificity at a given threshold for each study.

The threshold effect is a major source of heterogeneity among studies. Visual assessment of the ROC plane did not reveal significant threshold effect (Figure 5). Spearman correlation coefficient was 0.332 and the *p* value was 0.06, confirming no significant evidence of threshold effect.

As revealed by the Forest Plots of accuracy data, significant heterogeneity was detected according to the I^2^ values. Therefore, we performed a meta-regression to detect the source of heterogeneity analyzing the impact of country, study design, sample size, clinical stage, and detection methods. However, none of the above covariates contributed to heterogeneity (Table 2).

**TABLE 2:** Resuts of meta-regression analysis.

Variable	Coefficient	Standard error	*p* value^a^	RDOR	95% CI
Country	−0.077	0.0857	0.3742	0.93	0.78–1.10
Sample Size	0.000	0.0060	0.9404	1.00	0.99–1.01
Clinical Stage	−0.349	0.1732	0.0537	0.71	0.49–1.01
Detection Method	0.097	0.1444	0.5074	1.10	0.82–1.48

^a^ Tau-squared estimate.

**FIGURE 6:**
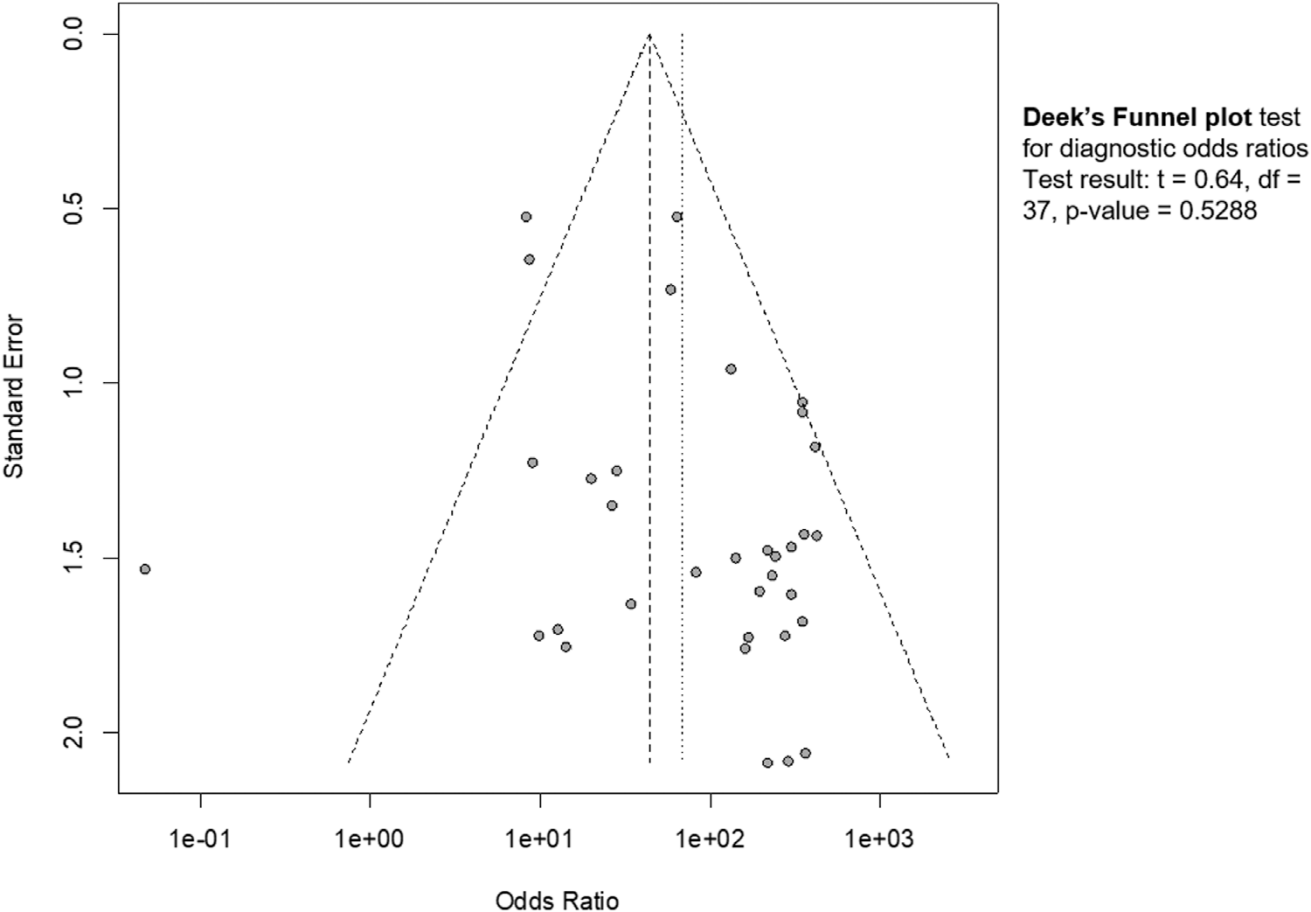
Funnel plot. The graphical representation showed no significant publication bias (*p* = 0.53).

Publication bias was estimated by funnel plot (Figure 6). The visual inspection revealed a partial symmetry with *p value* = 0.5288, showing no evidence of publication bias.

## Discussion

The need to identify a minimally invasive, efficient and repeatable over time approach to detect tumor mutations in NSCLC patients has always been the greatest challenge in the medical clinic. Serial sampling of traditional biopsies is in fact not feasible. Serial sampling may provide information on 1) relapse or progression of disease, 2) treatment response, 3) survival and 4) temporal and spatial tumor heterogeneity. These elements are of pivotal importance for the application of personalized therapies tailored to the patient’s genetic background. Recently, cfDNA analysis, namely “liquid biopsy”, has emerged as a non-invasive, feasible and reliable approach to investigate cancer DNA. However, its diagnostic accuracy for the detection of *KRAS* mutations remained controversial as different studies report varying degrees of sensitivity and specificity. As over one-third of lung adenocarcinomas harbour *KRAS* mutations, *KRAS* is therefore an important biomarker that might be used in monitoring treatment, as a biomarker for relapse/progression (disease monitoring), and as a prognostic biomarker.

We performed a large-scale meta-analysis of 40 papers and 2,805 NSCLC patients, reviewing controversial evidence for the diagnostic role of cfDNA testing for the detection of *KRAS* mutations in NSCLC. In our study, the pooled sensitivity for cfDNA was 0.71 (95% CI 0.68–0.74) and the specificity was 0.93 (95% CI 0.92–0.94) (Figure 2). None of the properties analyzed in the patient cohort appear to be specifically related to sensitivity. We are aware that the resulting sensitivity is not high enough as a diagnostic method. However, as a cancer screening test, if the test guarantees non-invasiveness, a high specificity is required instead of sensitivity. In this case, our meta-analysis identified high specificity, and AUC more than 0.9, indicating an overall high diagnostic efficiency in detection of *KRAS* mutations by cfDNA analysis (Figure 4). Also the values of the Likelihood ratios (PLR, and NLR) confirmed the informativeness of the test on cfDNA (Figure 3).

Conducting our meta-analysis, however, we ran into some noteworthy issues. First of all, there is high variability among clinical stages and consequently in the treatment among the 40 studies (Table 1). Second, different methods with different diagnostic performances were used to assess KRAS mutations in cfDNA in different studies highlighting the importance of method standardization. However, different studies using the same method have obtained diametrically opposite results from the point of view of diagnostic performance (see for example Leighl et al., 2019 and Jiao et al., 2021 both using Targeted NGS). This is due to the fact that numerous variables beyond the method affect the accuracy of mutation detection. Another issue is represented by the small size of some studies that might lead to bias. Last but not least, in most of the eligible studies, the analyzed tissues were formalin-fixed, paraffin-embedded (FFPE) which determine DNA degradation. All these factors represent important limitations that potentially increase the detection bias. Furthermore, it is not possible to overlook the difficulty in defining true negative (TN) or false positive (FP) when comparing detection rates of cfDNA analyses across different studies. A result can turn out to be “negative” in cfDNA if there really is no tumor DNA present or if tumor DNA is present but not detectable due to low amounts and the method is not sensitive enough to detect it. Similarly, mismatch between mutations found in cfDNA and mutations not found in tissue DNA does not necessarily imply FP in cfDNA. A false positive result in cfDNA could be due to DNA degradation of the tumor tissue; to a sampling problem in tissue; or to setting a limit of detection (LOD) threshold too low. Data on FP rates for each method and how the limit of detection (LOD) was determined should be available for each study so that they can be taken into account for cross-study comparison.

Significant heterogeneity was detected as revealed by the Forest Plots of accuracy data. However, Spearman Correlation and ROC plane plot (Figure 5) suggested that this heterogeneity was not caused by the threshold effect. The results of meta-regression showed that none of the analyzed variables was the source of heterogeneity (Table 2).

In conclusion, in the present meta-analysis of 40 studies including more than 2,800 NSCLC patients, on the basis of accumulated data, the detection of KRAS mutation in cfDNA proves to be of adequate diagnostic accuracy. This is a novel finding regarding KRAS since the previous published meta-analyses focused on the diagnostic role of EGFR detection. Targeted treatment against *KRAS* G12C is soon a reality, not only for NSCLC. For this reason, we need to search and find cancer patients with this mutation and we need sensitive and accurate methods for detecting *KRAS* in cfDNA. The present meta-analysis reveals that cfDNA analysis might be a valid alternative for molecular analysis when tumor biopsy or cytological specimens are not available. Given its non-invasive nature and the resulted high specificity, cfDNA testing represents a promising screening assay for detecting *KRAS* mutations in cancer patients.

## Data Availability

The original contributions presented in the study are included in the article/Supplementary Material, further inquiries can be directed to the corresponding author.
